# Outcomes of SARS‐CoV‐2 Infections in Patients with Neurodegenerative Diseases in the LEOSS Cohort

**DOI:** 10.1002/mds.28554

**Published:** 2021-02-27

**Authors:** Meret K. Huber, Claudia Raichle, Paul Lingor, Matthis Synofzik, Stefan Borgmann, Johanna Erber, Lukas Tometten, Wolfgang Rimili, Sebastian Dolff, Kai Wille, Samuel Knauss, Christiane Piepel, Julia Lanznaster, Siegbert Rieg, Fabian Prasser, Lisa Pilgram, Annika Spottke, Thomas Klockgether, Christine Klein, Franziska Hopfner, Günter U. Höglinger, the LEOSS Study Group

**Affiliations:** ^1^ Department of Neurology Hannover Medical School Hannover Germany; ^2^ Tropical Clinic Paul‐Lechler Hospital Tübingen Germany; ^3^ Department of Neurology, School of Medicine, Klinikum rechts der Isar Technical University of Munich Munich Germany; ^4^ German Center for Neurodegenerative Diseases (DZNE) Munich Germany; ^5^ Department of Neurodegenerative Diseases, Centre for Neurology and Hertie‐Institute for Clinical Brain Research University of Tübingen Tübingen Germany; ^6^ German Center for Neurodegenerative Diseases (DZNE) Tübingen Germany; ^7^ Department of Infectious Diseases and Infection Control Hospital of Ingolstadt Ingolstadt Germany; ^8^ School of Medicine University Hospital Rechts der Isar, Technical University of Munich Munich Germany; ^9^ Department I for Internal Medicine University Hospital of Cologne, University of Cologne Cologne Germany; ^10^ Department of Internal Medicine Stiftung Kreuznacher Diakonie Rhaunen Germany; ^11^ Department of Infectious Diseases University Hospital Essen, University of Duisburg‐Essen Essen Germany; ^12^ University Clinic for Hematology, Oncology, Hemostaseology and Palliative Care, Johannes Wesling Medical Center Minden, UKRUB University of Bochum Minden Germany; ^13^ Department of Neurology and Experimental Neurology Charité—Universitätsmedizin Berlin, Freie Universität Berlin, Humboldt Universität zu Berlin, Berlin Institute of Health Berlin Germany; ^14^ Department of Hemato‐Oncology and Infectious Diseases Klinikum Bremen‐Mitte Bremen Germany; ^15^ Department of Internal Medicine Hospital Passau Passau Germany; ^16^ Medical Center—University of Freiburg, Division of Infectious Diseases, Department of Medicine II, Faculty of Medicine University of Freiburg Freiburg Germany; ^17^ Charité—Universitätsmedizin Berlin, Freie Universität Berlin, Humboldt‐Universität zu Berlin, Berlin Institute of Health Berlin Germany; ^18^ Department of Internal Medicine, Hematology and Oncology Goethe University Frankfurt, Frankfurt am Main Germany; ^19^ German Center for Neurodegenerative Diseases Bonn Germany; ^20^ Department of Neurology University of Bonn Bonn Germany; ^21^ Institute of Neurogenetics University of Lübeck Lübeck Germany

The impact of preexisting neurodegenerative diseases on superimposed SARS‐CoV‐2 infections remains controversial. Here we examined the course and outcome of SARS‐CoV‐2 infections in patients affected by Parkinson's disease (PD) or dementia compared to matched controls without neurodegenerative diseases in the LEOSS (Lean European Open Survey on SARS‐CoV‐2‐infected patients) cohort, a large‐scale prospective multicenter cohort study.^1^

The LEOSS scientific data set comprises anonymous data after data quality control, including plausibility checks. Collected data include demographic information, standardized clinical classification of the SARS‐CoV‐2 severity (hospitalization and discharge), administered medical care (eg, intensive care unit [ICU] stay, and ventilation), preexisting and concomitant signs and symptoms, medication, laboratory parameters, and mortality. The patient sample age is grouped in decades.

Our study population comprised n = 4310 SARS‐CoV‐2‐infected patients (59% men). Forty of them had PD (median decade: 76–85 years, 63% men); 290 had dementia (median decade: 76–85 years, 50% men) (Supplementary Tables S1 and S2). Dementia was classified into Alzheimer's disease (22.1%), vascular dementia (13.3%), other dementia (12.4%), and unknown/missing value (52.1%). More than 95% of the patients were from tertiary referral centers in Germany between March 2020 and November 2020.

Using a systematic sampling strategy, we extracted 15 controls randomly from the study population for each PD patient (1:15) and 2 randomly selected controls for each dementia patient (1:2). Any potentially confounding effects resulting from variability in age and sex were fully adjusted for by the matching procedure. To avoid bias, we handled patients and controls the same way according to standard epidemiological principles.

The overall SARS‐CoV‐2‐associated mortality in the PD (32.5%) and dementia (32.1%) groups did not significantly differ from their respective control groups (28.7% and 26.5%).

Delirium occurred more frequently in dementia compared to PD and controls, but patient‐reported parameters (eg, dry cough and dyspnoe) were less frequent in dementia compared to PD and controls. Interestingly, dementia patients remained in the ICU and were ventilated for a shorter time period than controls. The major SARS‐CoV‐2 outcome parameters (duration of inpatient stay, duration of ICU stay, and duration of ventilation; SARS‐CoV‐2‐related mortality) were also not significantly different between PD patients, dementia patients, and controls. The age and gender distributions in our patient sample were not significantly different from previously published epidemiological cohort studies reporting the typical characteristics of German PD and dementia patients.^2^, ^3^, ^4^, ^5^ This suggests that our sample was representative of the patients observed in the general population. Only the subgroup of female dementia patients had a higher mortality than their female controls (Table 1).

**TABLE 1:** Parameters of SARS‐CoV‐2 disease course in patients with neurodegenerative comorbidity and controls

Parameter disease course	PD patients vs. controls		Dementia patients vs. controls		PD patients vs. dementia patients	
Duration of inpatient stay	*P* = 0.608	OR: NA*	*P* = 0.933	OR: NA*	*P* = 0.503	OR: NA*
Duration of ICU	*P* = 0.215	OR: NA*	***P* = 0.0003** shorter stay in ICU for D patients	OR: NA*	*P* = 0.899	OR: NA*
Ventilation duration	*P* = 0.256	OR: NA*	***P* = 0.0037** shorter ventilation for D patients	OR: NA*	*P* = 0.800	OR: NA*
Covid death	*P* = 0.605	OR 0.8347 CI [0.4208; 1.6556]	*P* = 0.084 men, *P* = 0.448 women, *P* = 0.00036 higher lethality for women patients with dementia vs. women controls	OR 0.7626 CI [0.5603; 1.0378]	*P* = 0.956	OR 1.02 CI [0.5034; 2.0664]
Death	*P* = 0.895	OR 0.955 CI [0.4821; 1.8922]	*P* = 0.057 men, *P* = 0.792 women, *P* = 0.0016 higher lethality for women patients with dementia vs. women controls	OR 0.7510 CI [0.5587; 1.0094] Men: OR 1.0563 CI [0.7025; 1.5883] Women: OR 0.4964 CI [0.3199; 0.7702]	*P* = 0.532	OR 0.7995 CI [0.3958; 1.6149]
Dry cough	*P* = 0.572	OR 1.237 CI [0.5914; 2.5877]	***P* = 0.00014** D patients with fewer dry cough	OR 2.0252 CI [1.4029; 2.9235]	*P* = 0.226	OR 1.6159 CI [0.7386; 3.5354]
Dyspnoe	*P* = 0.708	OR 0.8794 CI [0.4484; 1.7249]	***P* = 0.0085** D patients with fewer dyspnoe	OR 1.5743 CI [1.1211; 2.2107]	*P* = 0.100	OR 1.8008 CI [0.8854; 3.6624]
Fever	*P* = 0.194	OR 1.6 CI [0.783; 3.2677]	*P* = 0.247	OR 1.2006 CI [0.881; 1.6361]	*P* = 0.5439	OR 0.7935 CI [0.3788; 1.6624]
Delirium	*P* = 0.799	OR 0.7647 CI [0.0962; 6.0767]	***P* = 0.00056** D patients with more frequent delirium	OR 0.3125 CI [0.1563; 0.6249]	*P* = 0.223	OR 0.3028 CI [0.0396; 2.3156]
Headache	*P* = 0.423	OR 2.2348 CI [0.2971; 16.8076]	***P* = 0.00177** D patients with fewer headaches	OR 12.3931 CI [1.6674; 92.1096]	*P* = 0.117	OR 6.8718 CI [0.4212; 112.1193]
Taste disorder	*P* = 0.632	OR 1.6339 CI [0.2149; 12.4198]	***P* = 0.0342** D patients with fewer taste disorders	OR 4.3146 CI [0.9895; 18.8137]	*P* = 0.291	OR 3.4231 CI [0.3032; 38.6459]

Adjusted for age and sex. Univariate statistical analyses were performed to determine the significance between the analyzed subgroups. Odds ratios with the corresponding confidence intervals were generated. Abbreviations: PD, Parkinson's disease patients; D, dementia; controls, SARS‐CoV‐2 patients without comorbidities, Parkinson's disease or dementia; ICU, intensive care unit; OR, odds ratio; CI, confidence interval; OR, NA*, due to the data structure, multiple ORs are generated for the respective categories. These ORs can be obtained on request.

Although prior studies have reported higher SARS‐CoV‐2‐related mortality in patients with PD or dementia compared to patients without preexisting neurodegenerative diseases,^6^, ^7^ encouragingly, our comparably relatively large, well‐controlled, standardized data set with prospective patient enrollment does not support the notion of an increased risk for a fatal course of SARS‐CoV‐2 in PD or dementia patients, when treated in tertiary referral centers. Further research is required to shed light on the impact of gender on the outcome of SARS‐CoV‐2 infections in dementia patients.

## Ethics

Approval for LEOSS was obtained by the applicable local ethics committees of all participating centers and registered at the German Clinical Trials Register (DRKS, number S00021145).

## Author Roles

(1) Research project: A. Conception: F.H., M.K.H., P.L., M.S., C.R., A.S., T.G., C.K., G.U.H. B. Organization: F.H., M.K.H., P.L., M.S., A.S., T.G., C.K., G.U.H., C.R., L.P. C. Execution: M.K.H., C.R., P.L., M.S., S.B., J.E., L.T., W.R., S.D., K.W., S.K., C.P., J.L., S.R., F.P., L.P., A.S., T.K., C.K., F.H., G.U.H. (2) Statistical analysis: A. Design: F.H., M.K.H., P.L., M.S., A.S., T.G., C.K., G.U.H. B. Execution: F.H., M.K.H. C. Review and critique: F.H., M.K.H., P.L., M.S., S.K., A.S., T.G., C.K., G.U.H. (3) Manuscript preparation: A. Writing of the first draft: F.H., M.K.H., P.L., M.S., C.R., A.S., T.G., C.K., G.U.H. B. Review and critique: M.K.H., C.R., P.L., M.S., S.B., J.E., L.T., W.R., S.D., K.W., S.K., C.P., J.L., S.R., F.P., L.P., A.S., T.K., C.K., F.H., G.U.H.

## Financial Disclosures

Meret K. Huber, Claudia Raichle, Matthis Synofzik, Stefan Borgmann, Johanna Erber, Lukas Tometten, Wolfgang Rimili, Sebastian Dolff, Kai Wille, Samuel Knauss, Christiane Piepe, Julia Lanznaster, Siegbert Rieg, Fabian Prasser, Annika Spottke, and Thomas Klockgether report no disclosure. Paul Lingor is supported by the NUM research network of the BMBF (B‐FAST) and the Bavarian Staatsministerium für Wissenschaft und Kunst, and Lisa Pilgram is funded by the Willy Robert Pitzer Foundation. Christine Klein is supported by the B‐FAST Program (BMBF). Franziska Hopfner receives grants from the German Research Council (DFG), EASI‐Genomics Consortium (Horizon 2020), the Erwin‐Röver‐Foundation, and the Else Kröner‐Fresenius Foundation. Günter U. Höglinger is supported by the German Federal Ministry of Education and Research (BMBF: 01KU1403A EpiPD), Deutsche Forschungsgemeinschaft (DFG, German Research Foundation) under Germany's Excellence Strategy within the framework of the Munich Cluster for Systems Neurology (EXC 2145 SyNergy—ID 390857198), DFG grants (HO2402/6‐2, HO2402/18‐1 MSAomics), the NOMIS foundation (FTLD project), the EU/EFPIA/Innovative Medicines Initiative, Joint Undertaking (IMPRIND grant number 116060) and by the Volkswagen Stiftung/Lower Saxony Ministry for Science/Petermax‐Müller Foundation (Niedersächsisches Vorab ‐ Etiology and Therapy of Synucleinopathies and Tauopathies). All authors are government employees.

## Supporting information


**APPENDIX S1**. Supporting Information

## References

[1] Jakob CEM , Borgmann S , Duygu F , Behrends U , Hower M , Merle U , et al. First results of the “lean European open survey on SARS‐CoV‐2‐infected patients (LEOSS)”. Infection 2021;49:63–73. 10.1007/s15010-020-01499-0. 33001409 PMC7527665

[2] Enders D , Balzer‐Geldsetzer M , Riedel O , Dodel R , Wittchen HU , Sensken SC , et al. Prevalence, duration and severity of Parkinson's disease in Germany: a combined meta‐analysis from literature data and outpatient samples. Eur Neurol 2017;78(3–4):128–136. 10.1159/000477165 28746937

[3] Trenkwalder C , Schwarz J , Gebhard J , Ruland D , Trenkwalder P , Hense HW , et al. Starnberg trial on epidemiology of parkinsonism and hypertension in the elderly. Prevalence of Parkinson's disease and related disorders assessed by a door‐to‐door survey of inhabitants older than 65 years. Arch Neurol 1995;52(10):1017–1022. 10.1001/archneur.1995.00540340109020 7575219

[4] Bickel H . Demenzsyndrom und Alzheimer Krankheit: Eine Schätzung des Krankenbestandes und der jährlichen Neuerkrankungen in Deutschland. Das Gesundheitswesen 2000;62(04):211–218. 10.1055/s-2000-10858 10844818

[5] Jessen, F, Handbuch Alzheimer‐Krankheit: Grundlagen – Diagnostik – Therapie – Versorgung – Prävention, De Gruyter; 1. Edition, ISBN‐10: 3110403455

[6] Del Prete E , Francesconi A , Palermo G , Mazzucchi S , Frosini D , Morganti R , et al. Prevalence and impact of COVID‐19 in Parkinson's disease: evidence from a multi‐center survey in Tuscany region. J Neurol 2020. 10.1007/s00415-020-10002-6 PMC7471534 32880722

[7] Zhang Q , Schultz JL , Aldridge GM , Simmering JE , Narayanan NS . Coronavirus disease 2019 case fatality and Parkinson's disease. Mov Disord 2020;35(11):1914–1915. 10.1002/mds.28325 32954522 PMC7537245

