# Supplementary material for: Outcomes of SARS‐CoV‐2 Infections in Patients with Neurodegenerative Diseases in the LEOSS Cohort
Source: Mov Disord. 2021 Feb 27;36(4):791–3. doi: 10.1002/mds.28554 (PMC8014567; doi:10.1002/mds.28554)
Supplement: Supplementary file 1 — APPENDIX S1. Supporting Information [file MDS-36-791-s001.docx]

**Supplementary online material**

For data cleaning, data analysis and statistics we used Python (Python Software Foundation, <https://www.python.org/>), version 3.8.5final0. We extracted fifteen controls randomly from the study population for each PD patient (1:15) and two randomly selected controls for each dementia patient (1:2) by use of a systematic sampling strategy using python. Due to anonymous data collection, all metric data were recorded in categories with a temporal aggregation over the respective disease stages.

Table 1a. Parkinson's disease patients’ demographic data (absolute value)

| **Gender** | **Male** | **Female** | **All** |
| --- | --- | --- | --- |
| **Age** |  |  |  |
| **56-65** | 0 | 1 | 1 |
| **66-75** | 6 | 4 | 10 |
| **76-85** | 12 | 7 | 19 |
| **>85** | 7 | 3 | 10 |
| **All** | 25 | 15 | 40 |

Table 1b. Parkinson's disease patients’ demographic data (relative values, %)

| **Gender** | **Male** | **Female** | **All** |
| --- | --- | --- | --- |
| **Age** |  |  |  |
| **56-65** | 0.0 | 6.67 | 2.5 |
| **66-75** | 24.0 | 26.67 | 25.0 |
| **76-85** | 48.0 | 46.67 | 47.5 |
| **>85** | 28.0 | 20.00 | 25.0 |
| **all** | 100.0 | 100.00 | 100.0 |

Table 2a. Dementia patients’ demographic data, (absolute value)

| **Gender** | **Male** | **Female** | **All** |
| --- | --- | --- | --- |
| **Age** |  |  |  |
| **56-65** | 1 | 3 | 4 |
| **66-75** | 15 | 15 | 30 |
| **76-85** | 87 | 71 | 158 |
| **>85** | 42 | 56 | 98 |
| **All** | 145 | 145 | 290 |

Table 2b. Dementia patients’ demographic data, (relative values, %).

| **Gender** | **Male** | **Female** | **All** |
| --- | --- | --- | --- |
| **Age** |  |  |  |
| **56-65** | 0.69 | 2.07 | 1.38 |
| **66-75** | 10.34 | 10.34 | 10.34 |
| **76-85** | 60.00 | 48.97 | 54.48 |
| **>85** | 28.97 | 38.62 | 33.79 |
| **All** | 100.00 | 100.00 | 100.00 |
